# An empirical model to evaluate the effects of environmental humidity on the formation of wrinkled, creased and porous fibre morphology from electrospinning

**DOI:** 10.1038/s41598-020-74542-7

**Published:** 2020-11-02

**Authors:** Duo Zhang, Pooya Davoodi, Xia Li, Ye Liu, Wenyu Wang, Yan Yan Shery Huang

**Affiliations:** 1 Department of Engineering, University of Cambridge, Trumpington Street, Cambridge, CB2 1PZ UK; 2 The Nanoscience Centre, University of Cambridge, 11 JJ Thomson Ave, Cambridge, CB3 0FF UK

**Keywords:** Theory and computation, Chemical engineering, Design, synthesis and processing

## Abstract

Controlling environmental humidity level and thus moisture interaction with an electrospinning solution jet has led to a fascinating range of polymer fibre morphological features; these include surface wrinkles, creases and surface/internal porosity at the individual fibre level. Here, by cross-correlating literature data of far-field electrospinning (FFES), together with our experimental data from near-field electrospinning (NFES), we propose a theoretical model, which can account, phenomenologically, for the onset of fibre microstructures formation from electrospinning solutions made of a hydrophobic polymer dissolved in a water-miscible or polar solvent. This empirical model provides a quantitative evaluation on how the evaporating solvent vapour could prevent or disrupt water vapor condensation onto the electrospinning jet; thus, on the condition where vapor condensation does occur, morphological features will form on the surface, or bulk of the fibre. A wide range of polymer systems, including polystyrene, poly(methyl methacrylate), poly-l-lactic acid, polycaprolactone were tested and validated. Our analysis points to the different operation regimes associated FFES versus NFES, when it comes to the system’s sensitivity towards environmental moisture. Our proposed model may further be used to guide the process in creating desirable fibre microstructure.

## Introduction

Electrospinning is a versatile technique to generate fibres of diameters in the range of micrometer to tens of nanometers^1–3^. With respect to tuning fibre functionality, an ongoing interest is to control the fibre surface and internal microstructure at the individual fibre level, such as wrinkles, creases and porosity^4–6^. These localised, fibre microstructures could play important roles in modulating charge storage capability in batteries^7,8^, molecular absorption in sensors and filtration^9,10^, and cell-topography interaction in tissue engineering scaffolds^11,12^.

Solvent evaporation is a necessary step during solution-based electrospinning in air, hence the resulting fibre microstructures could be environmentally sensitive^13,14^. Relative humidity is known to be a key environmental factor influencing the fibre surface and internal microstructure. Within the scope of far-field electrospinning (FFES), a number of prior studies have presented systematic investigations into the interplay between relative humidity, solvent choice, and in some cases combined with temperature effect, for different polymer systems^13,14^. Although each of those previous studies provides great insight into the possible mechanisms leading to the fibre microstructure formation, the explanations and conclusions that they provided are specific to a particular polymer solution. Thus, it will be of general interest to utilise the existing experimental data available, to extrapolate a ‘rule-of-thumb’ for predicting the humidity effects on the fibre morphology produced by electrospinning.

Here, we propose a hypothesis which can account, phenomenologically, for the occurrence of fibre morphological features during electrospinning of a moisture-sensitive polymer solution. These electrospinning solutions should be formed by a hydrophobic polymer (< ~30 wt%) solubilised in a water-miscible or polar solvent. By cross-correlating data from far-field electrospinning (FFES), and near-field electrospinning (NFES), we propose an empirical model which can ‘index’ how a particular electrospinning setup can tolerate environmental moisture level. Once this setup-specific index is established, it can predict whether fibre morphological features will be resulted from electrospinning different polymer solutions under varied environmental humidity.

## Model hypothesis

### Examples from the classic PS solutions

**Figure 1:**
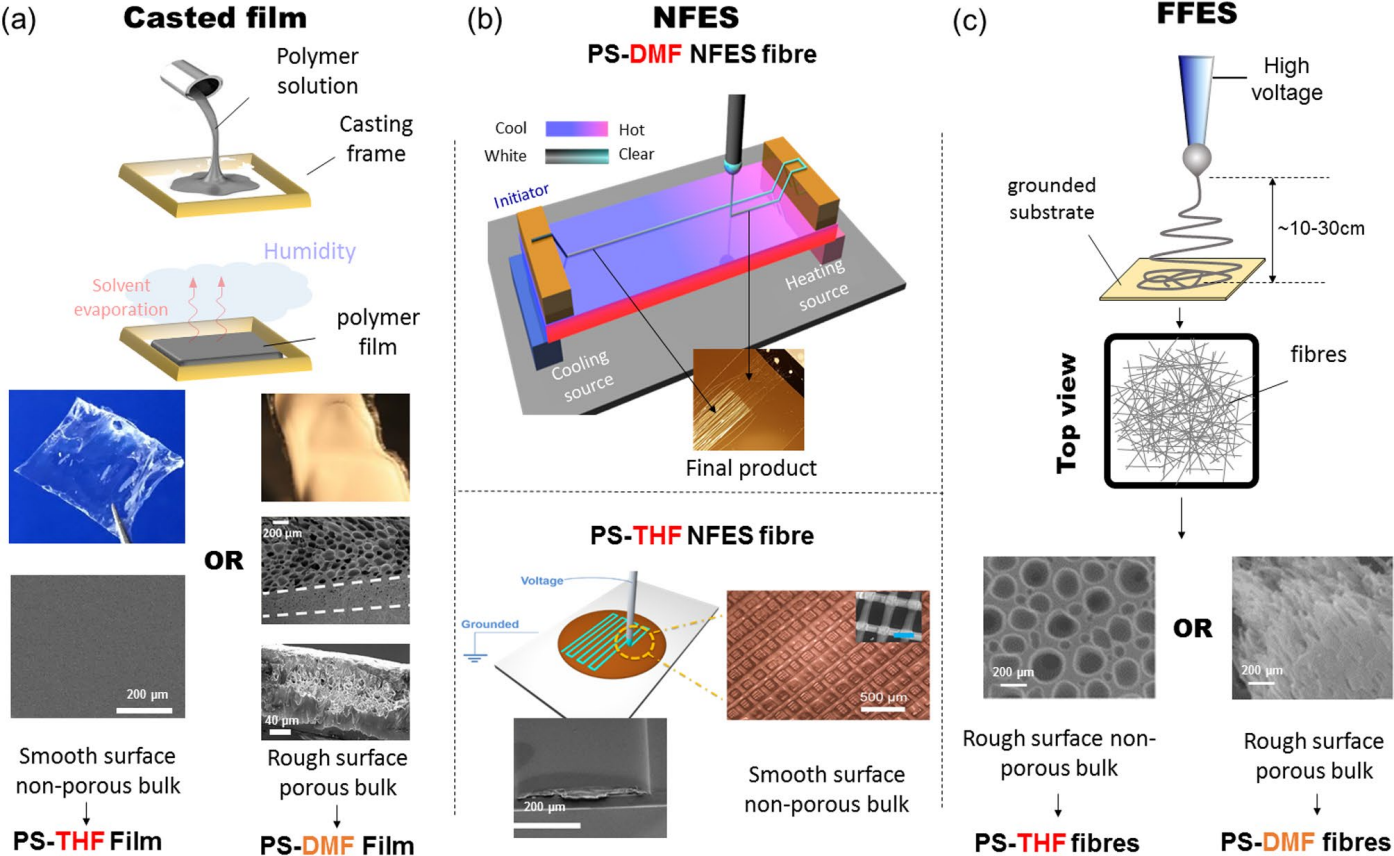
The schematic illustration of (**a**) film casting and (**b**) NFES and (**c**) FFES techniques used for the preparation of polymer films and microfibre structures. Micrograph images associated with FFES are extracted from Ref.^18^ (adapted with permission from American Chemical Society, Copyright 2013).

To establish a background to our hypothesis, we start by looking at classic electrospinning solution systems of PS-THF and PS-DMF (which both satisfy the previously prescribed polymer solution criteria). Polystyrene (PS) is typically considered as a hydrophobic polymer, where smooth, non-modified surfaces of PS typically has a water contact angle (WCA) of ~ 90°^15–17^. Both THF (boiling point = 66 °C) and DMF (boiling point = 153 °C) are polar solvents completely miscible with water at ambient conditions. Under typical FFES at ambient conditions (15–30 °C, relative humidity Hr >  ~20%), surface porosity is commonly observed on individual fibres produced from a PS-THF solution; in comparison, internal fibre inhomogeneity and porosity is commonly accompanied with the fibres produced from a PS-DMF solution. It is of interest to note that, performing film casting of the same PS-THF solution in similar environments will lead to a flat, smooth surface morphology of the film, unlike those produced from FFES. However, film casting of the same PS-DMF solution will lead to an opaque, strongly light-scattering appearance of the solidified film indicating internal structural inhomogeneity (Fig. 1).

**Figure 2:**
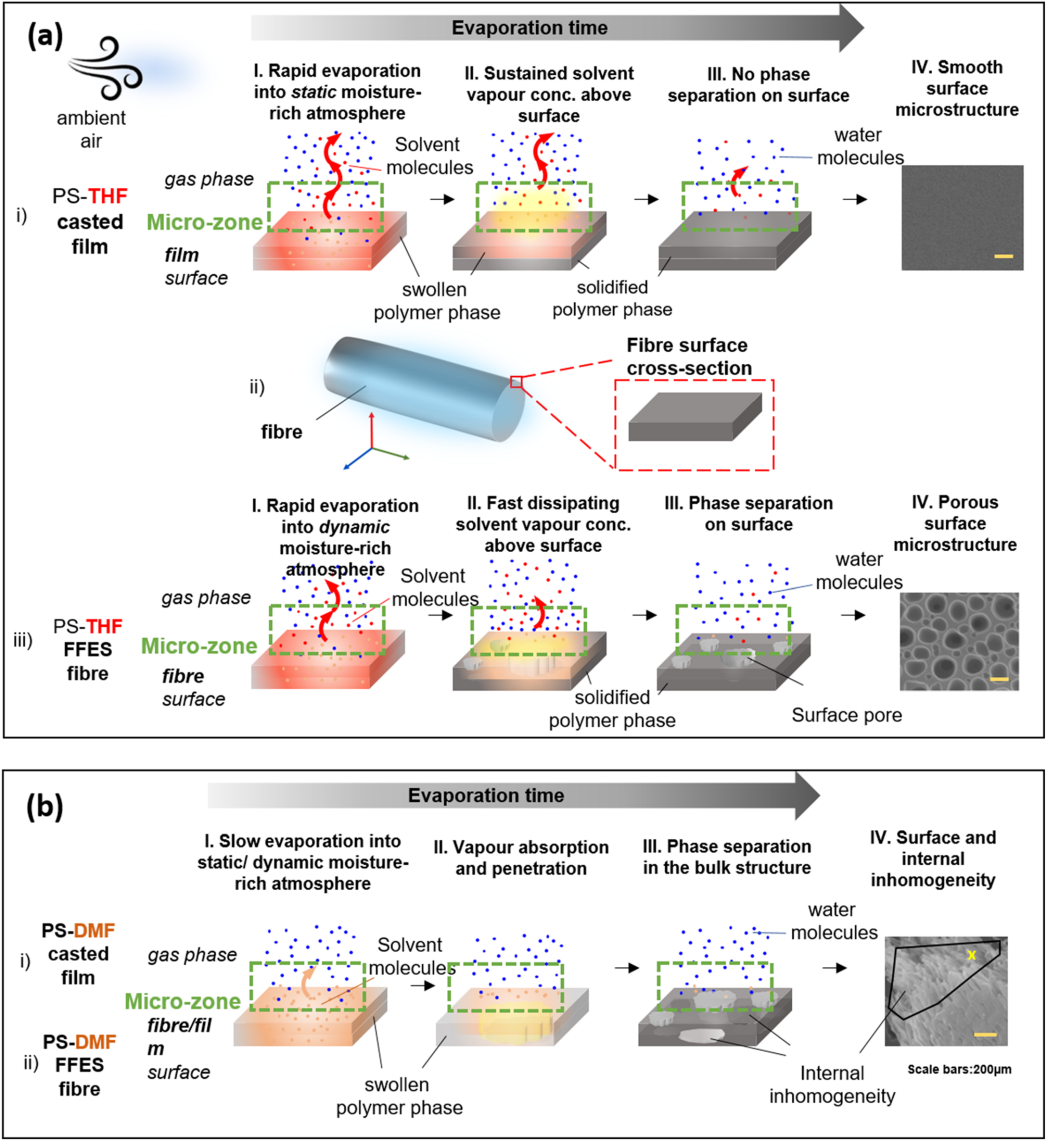
Proposed schemes demonstrating sequential stages of interaction between moisture (water vapour molecules) and solvent vapour molecules, at the air/substrate interface, for two polystyrene (PS) solution systems: (**a**) the PS-THF system, with (i) casted films accompanied by a smooth surface texture, and (ii, iii) fibres produced by far-field electrospinning (FFES), accompanied by a porous surface texture. (**b**) The PS-DMF system, where both (i) the casted film, and (ii) the FFES fibres exhibit similar surface and internal inhomogeneity^18^ (adapted with permission from American Chemical Society, Copyright 2013).

We hypothesize that the early-stage interaction between evaporated solvent molecules from the solvent-rich fibre surface, and the water molecules in air, plays an important role in determining the fibre surface morphology and/or internal fibre microstructure. Both the film and fibre fabrication techniques (FFES and NFES) incorporated in this study are presented in Fig. 1 and the interaction and microstructure are schematically presented in Fig. 2. In the early stages of evaporation, the polymer–solvent solution (swollen polymer phase) would consist of over 50% solvent composition. Different local concentrations of solvent or water vapour above the condensed phase/gas interface are presented depending on the humidity level and the solvent vapour pressure (of which are both temperature-dependent). The interaction at this ‘micro-zone’ region is dynamic and complex. However, from the aspect of whether the water vapour would condense and interact with the polymer solution surface (thus affecting the solidified polymer morphology), we propose that this probability is inversely proportional to the number density of solvent molecules in the micro-zone. The rationale is given by comparing the evaporation sequence for the PS-THF systems, between the casted film (Fig. 2a–i) versus the fibre fabricated via FFES (Fig. 2a–iii). THF is a low boiling point solvent (boiling point 66 °C), thus a fair amount of solvent vapour is expected to be presented in the micro-zone (shaded yellow in Stage I). It is expected that in the FFES scenario, when fibres are being electrospun and ejected out from the nozzle (Fig. 1c), the dynamic whipping motion of such solvent-rich fibres will be accompanied by a much faster evaporation and dissipation of the solvent vapour. The ‘interaction’ between water vapour molecules and solvent vapour molecules increases the chance of nucleation and condensation of water on the surface of FFES fibres. Although PS is a hydrophobic polymer, we suggest that PS-THF solution will have a better surface wetting property dominated by the presence of polar THF. Thus, the condensed water vapour from the fibre-surrounding atmosphere would be more liable to form condensation at the polymer fibres surface, rather than penetrating deep into the bulk of the fibres before complete solidification (from Stage I to Stage II). In comparison, a casted PS-THF film being placed within static moisture-rich atmosphere (Fig. 1a) would be dominated by much slower solvent diffusion outwards, and the inability to condense water would lead to smooth film surfaces on the transparent bulk structure (Fig. 2a–i). Moving on to the PS-DMF system (Fig. 2b), due to the high boiling point of DMF (153 °C), much less solvent vapour would be formed above the surface in both casted films and FFES fibres at room temperatures, giving water vapour a much longer time to condense on the polymer surface, and also penetrate into the bulk of the swollen polymer, causing a PS-DMF phase separation. The resulting fibre contains internal structures such as cavities (air pockets) and clusters; the surface will also contain creases or rough textures.

The above descriptions are qualitative in manner; and in line with the process phenomena described by Yazgan et al*.*^13^ The critical step relies on the ability of water to form condensed droplets on the swollen polymer surface within the micro-zone at Stage I (Fig. 2). Following this idea, we propose an empirical model, which can account for the transition (i.e. presence versus absence) in interfacial interaction at the water vapour and swollen polymer interface. This model can be applied to a broad range of polymeric solution systems, including highly hygroscopic solvents such as THF and DMF, extending to other solvents such as DCM and chloroform with limited water miscibility. It is important to note that our analysis is built based on the presumption that the polymer solution is already capable of forming fibres during an electrospinning process; thus our model proceeds to predict whether the moisture present in air would have sufficient influence on the fibre microstructure for the particular combination of polymer solution and electrospinning setup.

### Model description

Based on the above description, more solvent vapour in the micro-zone will lead to less probability for water vapour to form condensation nuclei. Therefore, we say the probability of water condensation is inversely proportional to the solvent vapour pressure. This solvent vapour pressure, $\overline{P}$ (in unit of Pascal, or J/m^3^) is approximated as scaling with the empirical Antoine relation (Eq. 1)^19^ where A, B and C are the known Antoine parameters for a particular solvent); *f* is a pre-factor (of which importance will be elucidated later).

1 $$\overline{P}\sim f10^{{\left( {A - \frac{B}{C + T}} \right)}}$$

The positive driving force for water to form condensate, *A*_*w*_, is assumed to be proportional to the relative humidity (*H*_*r*_), and the surface affinity of water *γ*_0_. It is important to note that, in our systems, we expect the variation in *γ*_0_ to be small provided that the solvent in the polymer solution has some water miscibility. As we will discuss, the surface affinity could influence the types of fibre morphological features, but not to shift the absence versus presence in the interaction between water vapour and the polymer solution surface. Further, the driving force, *A*_*w*_, should also go to zero at the freezing and boiling points of water at ambient pressure. Thus, we propose an empirical relation in the form of Eq. (2), with *T* being temperature in Kelvin. *k* is a normalisation factor.

2 $$ A_{w} \sim k\gamma_{0} H_{r} \left( {T - 273} \right)\left( {373 - T} \right) $$

Combining Eq. (1) and (2) leads to a parameter *C*_*w*_, measuring the propensity for water condensation

3 $$ C_{w} \sim \frac{{A_{w} }}{{\overline{P}}}\sim \frac{{k\gamma_{0} H_{r} \left( {T - 273} \right)\left( {373 - T} \right) }}{{f10^{{\left( {A - \frac{B}{C + T}} \right)}} }} $$

When $C_{w} \gg 1$, water vapour condenses severely and forms many inhomogeneities on the polymer surface or the bulk of the polymer after subsequent solvent evaporation. At the transition state, the condensed water vapor pressure and the evaporated solvent vapour pressure reach a dynamic balance. When $C_{w} \ll 1$, the polymer surface just becomes smooth. Thus the transition point can be set as $C_{w} \sim 1$. Since A, B and C are known Antoine constants for a particular solvent, and the ambient conditions in an experiment determines *H*_*r*_ and *T*, thus there is only one ensemble free parameter remaining, which is $\frac{{k\gamma_{0} }}{f}$. Here *k* is the normalization factor, and we expect the variation in *γ*_0_ to be small compared to *f*. Hence, *f* becomes the dominating free parameter to be evaluated for crossing over the transition point at $C_{w} \sim 1$. Empirically, we have found that assigning *kγ*_0_ ~ 1.4 will conveniently give *f* associated with $\overline{P}$ in the range of 0 and 1. Thus now, *f* would represent a concept similar to the ‘equivalent fraction of solvent vapour’ remaining close to the fibre/film surface to disrupt the water vapour condensation. Below, we will use different polymer–solvent cases from the literature data, and our own experiments, to illustrate how to deduce the parameter ‘*f*’ and proposing its significance.

One may postulate that the resulted fibre diameter influences the surface area to volume ratio, as well as the rate of evaporation. However, diameter is a derivative of the primary operating conditions (i.e. the solvent property of the electrospinning solution, the environmental conditions, and the electrospinning operating parameters), which are considered in the equations.

## Case studies of different polymer–solvent systems

In the laboratory, experiments are often conducted such that the same electrospinning setup is used to produce polymer fibres under varied solvent (Antoine parameters A, B, C known) and environmental conditions (i.e. varied and known humidity *Hr*, temperature *T*), and investigate how the different conditions affect fibre morphological features. For this reason, it is more convenient to compare the relative magnitudes of $A_{w} \sim k\gamma_{0} H_{r} \left( {T - 273} \right)\left( {373 - T} \right)\sim 1.4H_{r} \left( {T - 273} \right)\left( {373 - T} \right)$ versus $\overline{P}\sim f10^{{\left( {A - \frac{B}{C + T}} \right)}}$. Hence, if under a certain humidity and temperature condition, the electrospinning setup has a characteristic *f* leading to $A_{w} \ll \overline{P}\left( f \right)$, we will have the case of $C_{w} \ll 1$, where one expects minimal effective interaction between the ambient moisture and the polymer surface; and vice versus. One can establish *f* by looking at microscopic images of the fibre surface transition from the smooth condition ($A_{w} < \overline{P}\left( f \right))$, at the transition point ($A_{w} \approx \overline{P}\left( f \right)$), and then till the porous surface or internal inhomogeneity condition ($A_{w} > \overline{P}\left( f \right)$). Based on Eq. (3), then solving the equations would be able to find suitable values for *f*. Below, we will present examples for different polymer systems to illustrate the model’s applicability.

### Polystyrene system

**Figure 3:**
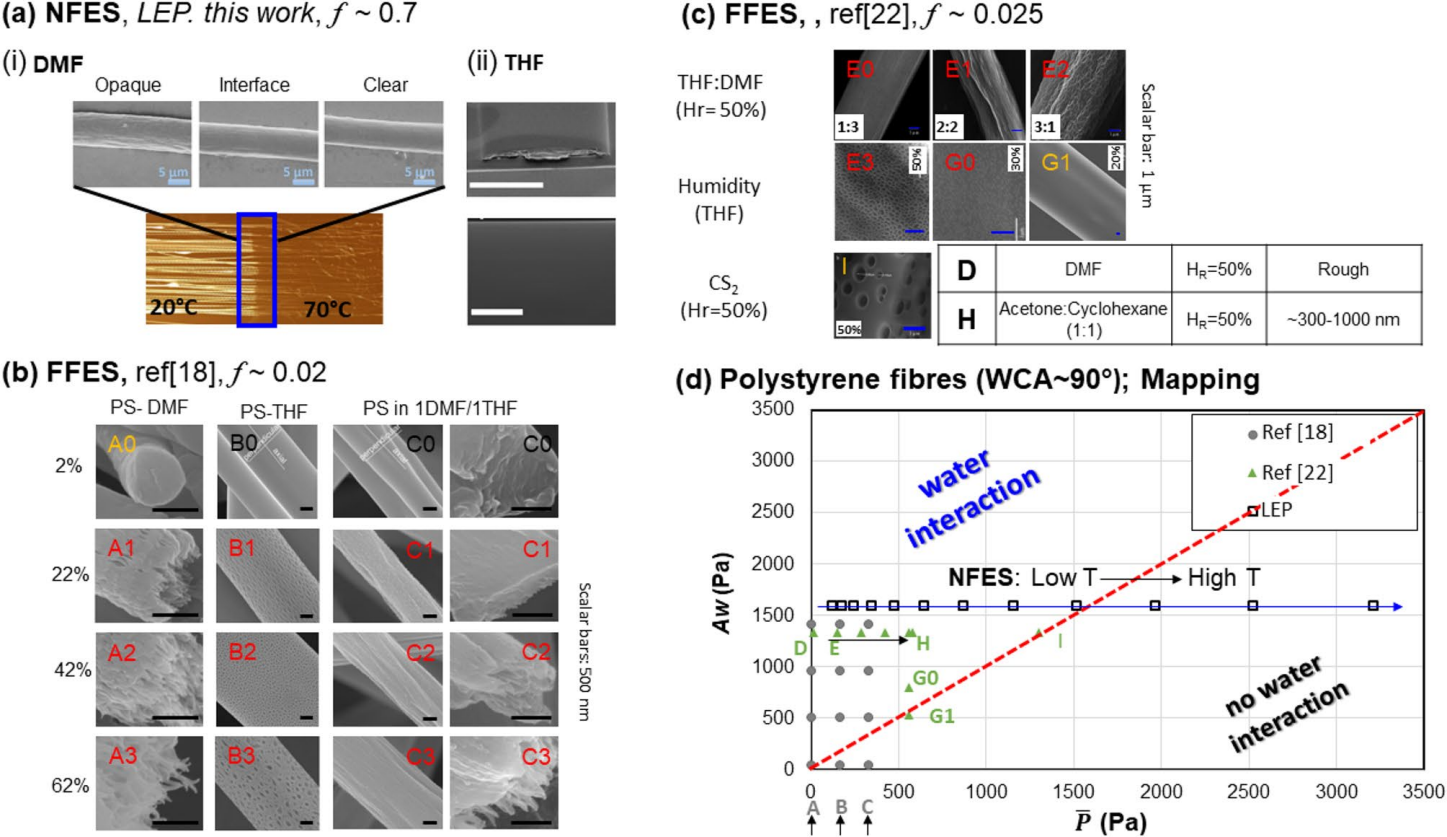
(**a**) Images illustrating different surface microstructures of NFES polystyrene (PS) fibres fabricated from (i) a PS-DMF solution system, and (ii) a PS-THF solution system. (**b**) Microstructures of PS fibres recaptured from Lu et al.’s FFES work^18^ (Adapted with permission from American Chemical Society. Copyright 2013). (**c**) Microstructures of PS fibres recaptured from Megelski et al.’s FFES work^20^ (Adapted with permission from American Chemical Society. Copyright 2002). In (**b** and **c**), each microstructure is labelled with an identifier, and are classified into one of three categories: with black-coloured labels indicating a smooth fibre surface, red labels a porous/textured fibre surface, and yellow-coloured label a transition (boundary). (**d**) Mapping of the estimated *A*_*w*_ versus $\overline{P}$ associated with different PS fibre fabrication conditions. Square symbols indicate values derived from conditions associated with NFES in (**a**); circular symbols from the FFES of (**b**); and triangular symbols from FFES of (**c**). WCA = water contact angle. (Note: The dotted red-line indicates the transition condition of $A_{w} \approx \overline{P}$ . If we cross this line from to the left-hand-side to the right-hand-side, we will cross the water interaction area to no water interaction area).

Here, we will examine the above model based on different polystyrene solutions tested in different FFES and NFES experimental setups, by fitting a single *f* parameter in each setup. Firstly, for the NFES from our own work, a PS-DMF solution is used to pattern fibres on a substrate maintained with a temperature gradient along the fibre-axis, shown in Fig. 3a,i. This yields a visually sharp transition in the fibre longitudinal opaqueness at the position point where the substrate temperature is ~ 50 °C. With the environmental conditions of *H*_*r*_~70% and 20 °C above the heating substrate, *A*_*w*_ ~ 1600. Taking the fibre microstructure transition occurring at a temperature similar to the substrate temperature *T* ~ 50 °C in Fig. 3b, solving $\overline{P}\left( f \right)\sim A_{w} \sim 1600$, will give *f* ~ 0.7. The graphical representation of $\overline{P}\left( f \right)\sim A_{w} \sim 1600$ is shown as the blue line in Fig. 3d. This line would cross the $A_{w} \sim \overline{P}$ transition line at $\overline{P}_{DMF}$ (T ~ 50 °C). Thus, for the left-side region of the transition line, it predicts water interaction, and thus we expect to observe fibre inhomogeneity; while for the right-side region of the transition line, it predicts no water interaction, and we expect to see a clear fibre. Hence, the NFES setup utilised in our work is characterised by a system parameter of *f* ~ 0.7. With this knowledge, we use a different solvent, based on THF to evaluate our model’s predictive power. Using *f* = 0.7 for the same NFES setup, all the $\overline{P}_{THF}$ values would lie on the right-side region of the transition line. This predicts that only smooth, clear fibres would be produced; and indeed this was expected for NFES of PS-THF solution in our setup, as demonstrated in Fig. 3a, ii.

Next, we will evaluate our model against Lu and Xia ’s study^18^, which presents how humidity and the molar ratio of THF:DMF modify the fibre microstructure for their FFES setup. The extracted scanning electron microscope (SEM) images are illustrated in Fig. 3b. Based on Raoult’s Law for ideal mixture of liquids, we determine the co-solvent vapour pressure following the assumptions that $\overline{P}_{xDMF:yTHF} = f\left( {xP_{DMF} + yP_{THF} } \right)$, where *x:y* is the DMF to THF molar ratio. We then evaluate the values of $\overline{P}_{xDMF:yTHF}$ with respect to *A*_*w*_ for a particular experimental humidity condition (Table S1). After comparing the calculated $\overline{P}_{xDMF:yTHF}$–*A*_*w*_ pairs associated with the experimental conditions which produced the fibre textures (see micrographs A0 to C3), *f* is expected to lie within the range of 4.8509 × 10^−3^ ~ 0.0274. We further fix *f* to a single value of *f* = 0.02 for this study, allowing point A0 plotted on Fig. 3d to lie close to the transition line $A_{w} \sim \overline{P}$. With this, we see that, while points A1–A3, B1–B3, and C1–C3 all lie in the ‘water interaction’ region, points B0 and C0 lie in the ‘no water interaction’ region. The above mapping thus reproduces the condition-dependent microstructure seen in Fig. 3b.

In the final example of the PS system, we use data from Megelski et al.’s work^20^ which investigated how the FFES PS fibre microstructures were affected by a variety of solvent and environmental conditions, in Fig. 3c. Using the afore-mentioned approach to include the experimental conditions of E0 to G1 into our model, we get the range of *f* to 0.027 to 0.11946. By setting *f* = 0.027 to allow G1 point lying near the transition line, we see that the corresponding *A*_*w*_ versus $\overline{P}$ data points (D to I) in Fig. 3d again reproduce the fibre microstructure for all the conditions considered. It is worth pointing out that, point I and point H were based on the CS_2_ and Acetone: Cyclohexane co-solvent system respectively. These two solvents have only limited water solubility, unlikely the complete water miscible natures of THF and DMF. Therefore, our model was proven to be applicable for different solvent systems, provided there is limited water solubility.

### PCL and PMMA systems

**Figure 4:**
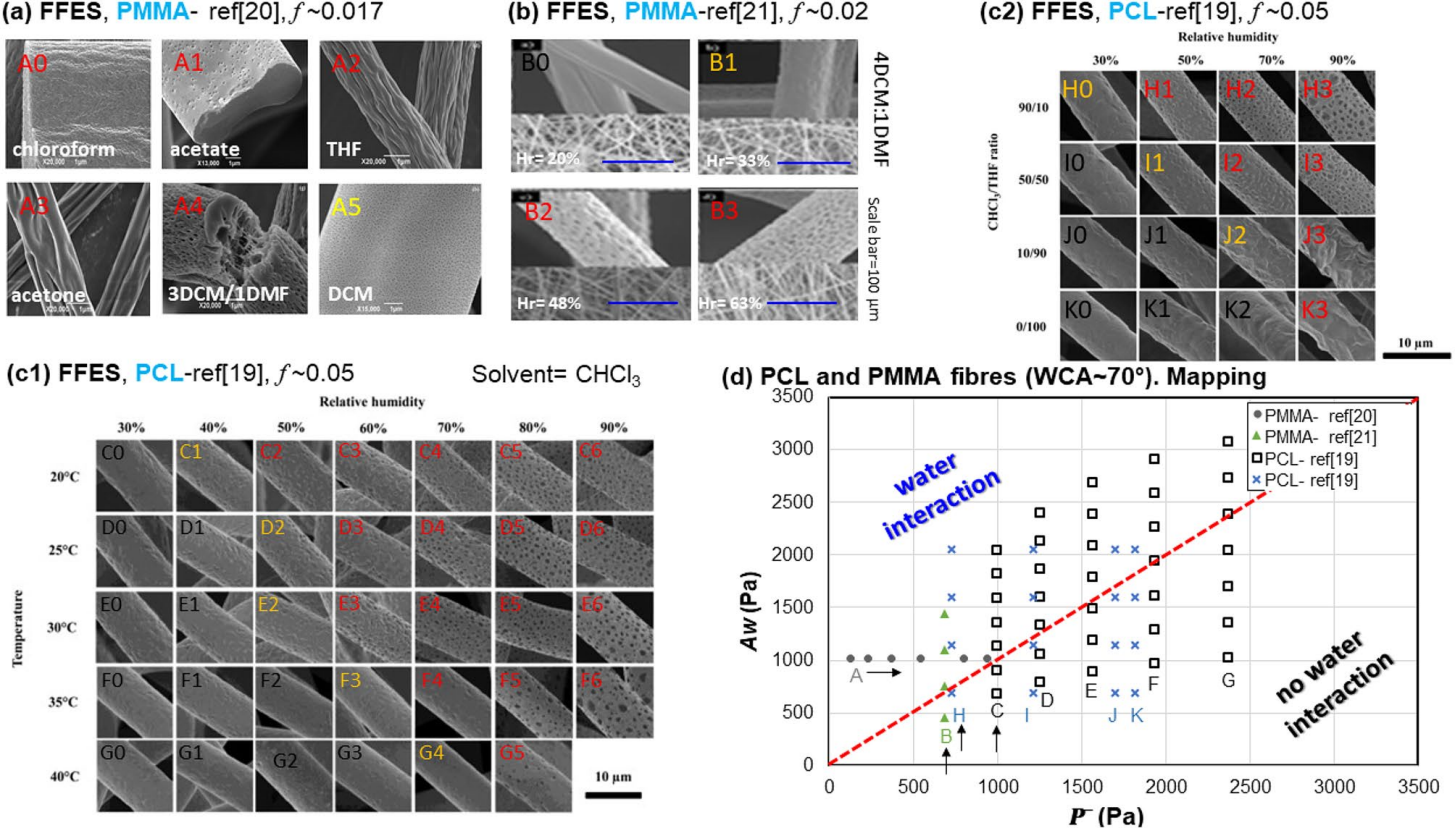
(**a**) Microstructures of PMMA fibres recaptured from Li et al.’s FFES work^23^. (Adapted with permission from Royal Society of Chemical, Copyright 2014). (**b**) Microstructures of PMMA fibres recaptured from Bae et al.’s FFES work^24^ (Adapted by permission from Springer Nature, Copyright 2013). (**c1** and **c2**) Microstructures of PCL fibres recaptured from Putti et al.’s FFES work^25^ (Adapted by permission from Elsevier, Copyright 2015). (**d**) Mapping of the estimated *A*_*w*_ versus $\overline{P}$ associated with PMMA or PCL undergoing different FFES conditions. The circular symbols indicate values derived from experimental conditions of (**a**); triangular symbols from conditions of (**b**); square symbols from conditions of (**c1**); and cross symbols from conditions of (**c2**). The dotted red-line indicates the transition condition of $A_{w} \approx \overline{P}$.

In this section, we evaluated the applicability of our model beyond the hydrophobic PS system. Both PCL and PMMA have a WCA of ~ 70°^21,22^, thus their electrospun solution systems are jointly studied here. First, Li et al.’s work^23^ investigates a range of single and co-solvents for FFES of PMMA fibres at a fixed humidity and temperature (Fig. 4a). It is to note that these solvent systems have very different water miscibility. From J0 to J1, the sequence of solvents used have an increased $\overline{P}$ values. Applying the above method we obtain *f* in the range of 0 to 0.0175; Setting *f* = 0.017 for their FFES setup, we see the corresponding *A*_*w*_ versus $\overline{P}$ data points in Fig. 4e all lie in the water-interaction region, with J5 close to the transition line. As shown in Fig. 4a, all the fibre microstructure display surface porosity indicating moisture interaction during the electrospinning process. Secondly, Bae et al.^24^ investigated a 4DCM:DMF (molar ratio) co-solvent system for FFES of PMMA under different humidity conditions (see Table S1). In this case *f* is within the range of 0.011 and 0.028; Using *f* = 0.02, the data points K0–K3 plotted in Fig. 4e clearly shows a transition from lying in the no water interaction region (K0), to near the transition line (K1), to lying in the water interaction region (K2, K3). These are again reflective of the associated SEM shown in Fig. 4b.

Moving onto FFES of PCL fibres, Fig. 4c1 and Fig. 4c1 comes the same paper, which provides systematic studies on firstly, the interplay of temperature and humidity on the PCL-THF system (Fig. 4c1); and secondly, the interplay between humidity and co-solvents (Fig. 4c2). Despite the complexity of the two experimental settings, cross-correlating the stated environmental and solvent conditions with the micrographs will give the ranges of *f* values associated with each figure overlapping. In other words, this confirms that *f* can be used to characterise a particular electrospinning setup. Additional analysis was also performed for the work of Yazgan et al. on PCL and PLLA with different experimental conditions, estimating *f* ~ 0.17 for their FFES setup. All data in Fig. 4a–c have been summarised in Fig. 4d which is mapping of the estimated *A*_*w*_ versus $\overline{P}$ associated with PMMA or PCL undergoing different FFES conditions. The corresponding micrographs and calculations can be found in the Supporting Information.

## Parameter *f* as a moisture-tolerance index

**Figure 5:**
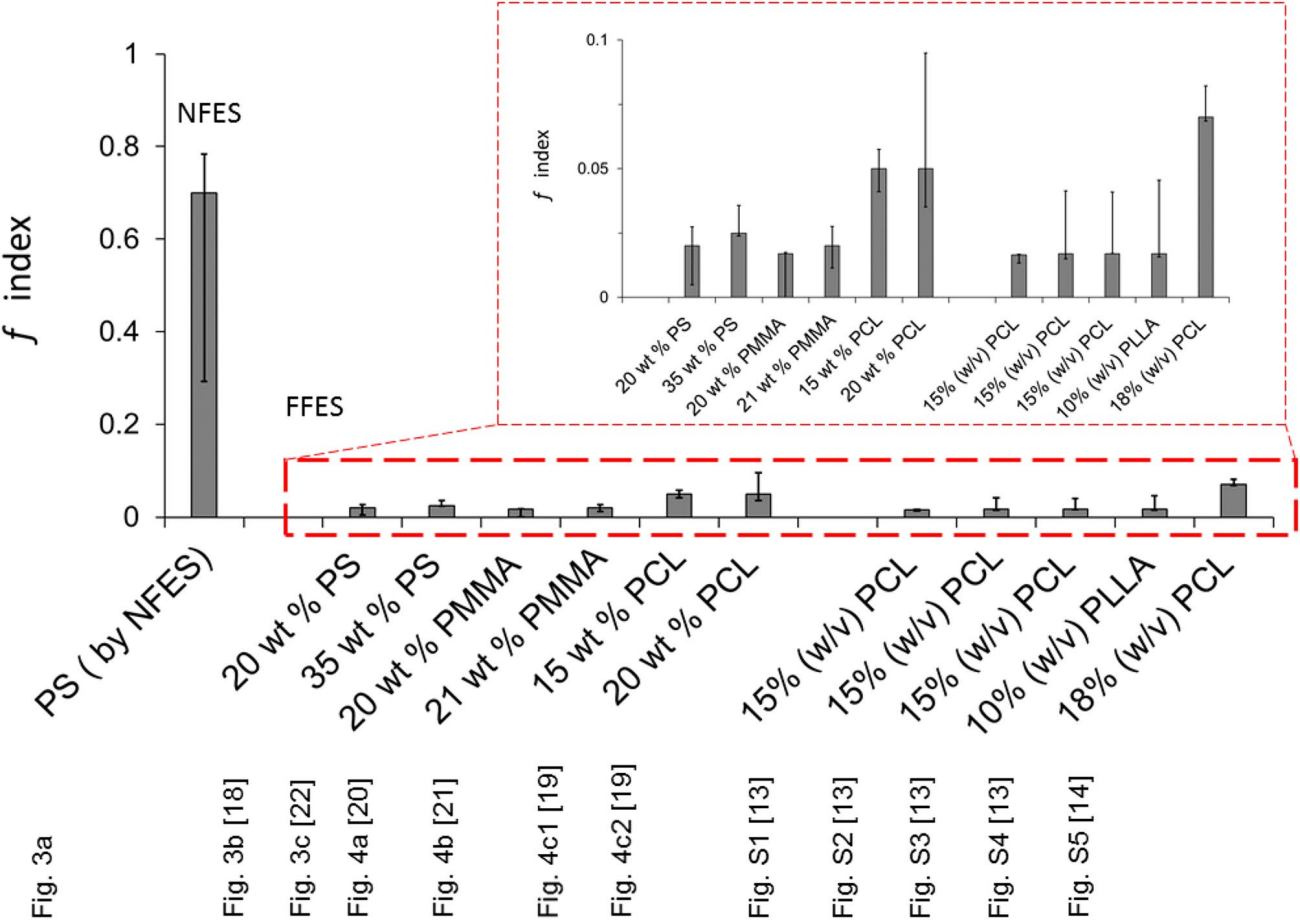
Summary of *f* values for all the experimental work quoted in this study. In the bar chart, the bounds indicate the upper and lower ranged values, where the chosen value used to evaluate the fibre microstructure is indicated as the bar-value. The FFES are further zoomed-in in the insert.

Overall, our hypothesis based on Fig. 2, was able to reproduce an extensive set of experimental observations, by fitting the free-parameter *f* in Eq. 3 for a particular electrospinning system. In the previous section, we elucidated *f*, as an ‘equivalent fraction of solvent vapour’ remaining to prevent or disrupt the water vapour condensation. The range of *f* values associated with different studies are summarised in Fig. 5. We propose that an alternative, more application-focused concept is to view *f* as an index characterising the electrospinning setup providing intrinsic tolerance to moisture affecting fibre morphology. From Fig. 5, we can see the fitted *f* values for the various FFES studies lie between 0.017 to 0.05 (thus, highly sensitive to environmental moisture); in fact, while four independent studies have *f* < 0.03, the two FFES studies shown in Fig. 4c1–c2 with *f* = 0.05 actually blow chloroform vapor into their atmospheric control chamber^25^. This can explain the relatively higher fitted *f* values in the work of Putti et al*.*^25^ in comparison to other FFES cases. In comparison, the *f* values associated with NFES is much greater; in our setup, *f* ~ 0.7 (thus has comparatively good moisture tolerance). The high *f* value associated with a NFES setup also means that it would be difficult to form surface porosity based on the mechanism of water vapor-induced phase separation (VIPS)^18^ utilised for FFES.

## Conclusions

We proposed a generic model which accounts for the formation of surface and internal fibre microstructures as a result of moisture interaction during electrospinning. This model was evaluated against a number of polymer systems based on PS, PCL, PLLA, and PMMA with their respective electrospinning solutions. Although the fabrication of electrospinning fibres could vary significantly across different laboratory settings, our model successfully integrated the varied experimental factors into one ensemble, free fitting parameter *f*, to predict the onset of water condensation interaction with solvent-rich fibres. The parameter *f* can be treated as an ‘equivalent fraction of solvent vapour’ remaining to prevent or disrupt the water vapour condensation. It is to note that some pre-assumptions have already been made for our model’s validity: firstly, the investigation is automatically limited to the solutions which can produce continuous electrospun fibres; secondly, the polymers should be hydrophobic in nature (as a pre-requisite for phase separation); and thirdly, the solvent should have some miscibility with water. The third criteria also means that we exclude the consideration for water immiscible solvents such as toluene and hexane (which are expected to produce smooth fibres). We found that a low value of *f* is a characteristic of FFES, and *f* close to 1 is typical of NFES. In other words, NFES is less environmentally sensitive to moisture. The notable difference in *f* values between the two methods may be a result of the difference in mechanical stretching experienced, where the FFES fibres would be subjected to greater mechanical stretching leading to higher probabilities for moisture condensation. The fitted parameter *f* could provide a calibration factor for a particular electrospinning setup, which can be used to predict, for example, the feasibility in forming surface porous fibre structures via VIPS for new polymer–solvent systems.

## Supplementary information

Supplementary information.

Supplementary Figure 3.

Supplementary Figure 4.

## Abbreviations

THF: Tetrahydrofuran
DMF: *N,N,*-Dimethylformamide
PS: Polystyrene
FFES: Far-field electrospinning
NFES: Near-field electrospinning
WCA: Water contact angle
DCM: Methylene dichloride
PCL: Polycaprolactone
PMMA: Poly methyl methacrylate
PLLA: Poly-l-lactic acid
VIPS: Vapor induced phase separation
CS_2_: Carbon disulfide
