# Supplementary material for: An empirical model to evaluate the effects of environmental humidity on the formation of wrinkled, creased and porous fibre morphology from electrospinning
Source: Sci Rep. 2020 Nov 2;10:18783. doi: 10.1038/s41598-020-74542-7 (PMC7608675; doi:10.1038/s41598-020-74542-7)
Supplement: Supplementary file 1 — Supplementary information. [file 41598_2020_74542_MOESM1_ESM.docx]

# Supplementary Information

**An Empirical Model to Evaluate the Effects of Environmental Humidity on the Formation of Wrinkled, Creased and Porous Fibre Morphology from Electrospinning**

Yan Yan Shery Huang^1,2*^, Duo Zhang^1,2^, Pooya Davoodi^1,2^, Xia Li^1^, Ye Liu^1,2^, Wenyu Wang^1,2^

^1^Department of Engineering, Trumpington Street, CB2 1PZ, United Kingdom

^2^The Nanoscience Centre, University of Cambridge, 11 JJ Thomson Ave, Cambridge CB3 0FF, United Kingdom

^*^Corresponding Author

Email: [yysh2@cam.ac.uk](mailto:yysh2@cam.ac.uk) (Y.Y.S. Huang)

**Table S1**. Polymer - solvent experiments

| **Examples*** | **Fiber polymer & Solvents** | **Relative Humidity** | **Temperature** | **Pre-factor *f*** |
| --- | --- | --- | --- | --- |
| **1. Fig. 3a** | **Polystyrene (PS) -- DMF** | ~ **70%** | **20 °C**  **(Fiber: 10-70°C)** | ***f* = 0.7 (0.2927 ~ 0.78409)** |
| **2. Fig. 3b [18]** | **20 wt % PS -- DMF;**  **20 wt % PS -- THF;**  **20 wt % PS -- DMF/THF (1:1 w/w)** | **2-62%** | **20 °C** | ***f* = 0.02 (4.8509×10^-3^ ~ 0.02735)** |
| **3. Fig 3c [22]** | **35 wt % PS -- THF**  **35 wt % PS -- THF/DMF (75/25 v/v)**  **35 wt % PS -- THF/DMF (50/50 v/v)**  **35 wt % PS -- THF/DMF (25/75 v/v)** | **20-70%** | **75-76 °F (24°C)** | ***f* = 0.025**  **(0.02381~0.0**3571**)** |
| **4. Fig 4a [20]** | **20 wt % PMMA -- Chloroform**  **20 wt % PMMA -- Ethyl Acetate**  **20 wt % PMMA -- THF**  **20 wt % PMMA -- Acetone**  **20 wt % PMMA -- DCM/DMF (3:1 v/v)**  **20 wt % PMMA -- DCM** | **38% ± 2** | **298 ± 2 K (25°C)** | ***f* = 0.017 (0 ~ 0.01742)** |
| **5. Fig. 4b [21]** | **21 wt % PMMA -- DCM/DMF (4:1 v/v)** | **15 -70%** | **Room temperature (25°C)** | ***f* = 0.02 (0.0114 ~ 0.0275)** |
| **6. Fig. 4c1 [19]** | **15 wt % PCL-- CHCl_3_** | **30 -90%** | **20°C-40°C** | ***f* = 0.05 (0.04111 ~ 0.05756)** |
| **7. Fig. 4c2 [19]** | **15 wt % PCL -- CHCl_3_/THF (90/10 w/w)**  **20 wt % PCL -- CHCl_3_/THF (50/50 w/w)**  **20 wt % PCL -- CHCl_3_/THF (10/90 w/w)**  **20 wt % PCL -- THF** | **30 -90%** | **20°C** | ***f* = 0.05 (0.03506 ~ 0.05844)** |
| **8. Fig. S1 [13]** | **15% (w/v) PCL -- DCM**  **15% (w/v) PCL -- CHCl_3_** | **12-55%** | **20°C** | ***f* = 0.0165 (0.01323 ~ 0.01675)** |
| **9. Fig. S2 [13]** | **15% (w/v) PCL-- CHCl_3_/MeOH (18/2 v/v)**  **15% (w/v) PCL -- CHCl_3_/DMSO (18/2 v/v)** | **12 -55%** | **20°C** | ***f* = 0.017 (0.01488 ~ 0.04128)** |
| **10. Fig. S3 [13]** | **15% (w/v) PCL -- CHCl_3_/DMSO (16/4 v/v)**  **15% (w/v) PCL-- CHCl_3_/DMSO (19/1 v/v)** | **12% -55%** | **20°C** | ***f* = 0.017 (0.01696 ~ 0.04088)** |
| **11. Fig. S4 [13]** | **10% (w/v) PLLA - CHCl_3_/DMF (17/3 v/v)** | **12 -55%** | **20°C** | ***f* = 0.017 (0.01562 ~ 0.04555)** |

*For references, please refer to the main manuscript.

**Supplementary Figures**

#
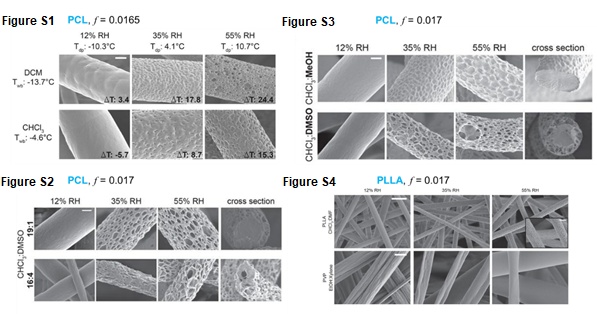


**Figure S1-S4.** PCL and PLLA microfibres fabricated in different conditions (Reproduced from Ref. [13]).

**Calculation process for evaluating the range of pre-factor** $\boldsymbol{f}$

As for the in-air water vapour pressure, $A_{w}\sim P/{P_{0}\sim}k{\gamma_{0}H}_{r}\left( T-273 \right)\left( 373-T \right)$ eq. [2].

As discussed in the “model description” part in the main text, is assigned as $k{\gamma_{0}\sim1.4}$, so eq. [2] could be simplied as:

$$A_{w}\sim P/{P_{0}\sim}1.4H_{r}\left( T-273 \right)\left( 373-T \right)$$

The Antoine equation is a class of semi-empirical correlations describing the relation between vapor pressure and temperature for pure components. The Antoine equation is:

$$\log_{10}(P)=A-\frac{B}{C+T}$$

where *P* is the vapour pressure, *T* is the temperature and *A*, *B*, *C* are component-specific constants.

**Figure 3a**

Relative humidity *RH*=70%; since there is no closed temperature chamber, the in-air water vapour temperature is supposed to be the room temperature 20°C (293.15 K).

For the in-air water vapour pressure, based on the Eq. 2

$$A_{w} (@ RH 70\%, 20^{\circ}C)\sim1.4\times0.7\times\left( 293.15-273 \right)\times\left( 373-293.15 \right)$$

For DMF, the Antoine parameters are *A*=7.24128, *B*=1597.92, *C*=213.457

Antoine Equation: $\log_{10}(P)=A-\frac{B}{C+T}$

Here *P* is the vapour pressure (unit in *mmHg*), *T* is the temperature (unit in °C); 1 *mmHg* =133.32237 Pa

So $f=0.7$ is set in this experimental case. $4.8509\times10^{-3}\leq f\leq0.0274$

**Figure 3b**

Relative humidity was changing at different levels; the experiments were conducted at a fixed temperature 20°C (293.15 K), thus the in-air water vapour temperature was 20°C (293.15 K).

For the in-air water vapour pressure, based on the Eq. 2:

$$A_{w} (@ 20^{\circ}C)\sim1.4\times RH\times\left( 293.15-273 \right)\times\left( 373-293.15 \right)$$

For DMF, the Antoine parameters are *A*=7.24128, *B*=1597.92, *C*=213.457

For THF, the Antoine parameters are *A*=7.10537, *B*=1256.68, *C*=232.621

Antoine Equation: $\log_{10}(P)=A-\frac{B}{C+T}$

Here *P* is the vapour pressure (unit in *mmHg*), *T* is the temperature (unit in °C); 1 *mmHg* =133.32237 Pa.

1) For DMF, A0 is at the boundary conditions, as the wrinkled surface could be seen on the surface of the fibers; A1 is at the water-interaction condition, as the surface pores and internal inhomogeneity could be seen.

2) For THF, B0 is at the no-water-interaction condition, as the fibre surface was smooth; B1 is at the water-interaction condition, as the surface pores could be seen.

3) For DMF/THF (1:1, wt), B0 is at the no-water-interaction condition, as the fibre surface was smooth; B1 is at the water-interaction condition, as the surface pores could be seen. Also Raoult’s Law for ideal mixture of liquids was applied, taking co-solvent vapour pressure to be $\bar{P}_{xDMF:yTHF}=f(xP_{DMF}+yP_{THF})$ , where *x:y* is the DMF to THF molar ratio, we can evaluate $\bar{P}_{xDMF:yTHF}$ with respect to $A_{w}$ for a particular experimental humidity condition.

So in total $4.8509\times10^{-3}\leq f\leq0.0274$, and$f=0.02$ is set in this experimental case.

**Figure 3c**

Relative humidity was changing at different levels; the experiments were conducted within the temperature 75°F -76°F, thus the in-air water vapour temperature was supposed as 24°C (297.15 K).

For the in-air water vapour pressure, based on the Eq. 2

$$A_{w} (24^{\circ}C)\sim1.4\times RH\times\left( 297.15-273 \right)\times\left( 373-297.15 \right)$$

For DMF, the Antoine parameters are *A*=7.24128, *B*=1597.92, *C*=213.457

For THF, the Antoine parameters are *A*=7.10537, *B*=1256.68, *C*=232.621

Antoine Equation: $\log_{10}(P)=A-\frac{B}{C+T}$

Here *P* is the vapour pressure (unit in *mmHg*), *T* is the temperature (unit in °C); 1 *mmHg* =133.32237 Pa.

1) For DMF/THF mixtures, Raoult’s Law for ideal mixture of liquids was applied, taking co-solvent vapour pressure to be $\bar{P}_{xDMF:yTHF}=f(xP_{DMF}+yP_{THF})$ , where *x:y* is the DMF to THF molar ratio, we can evaluate $\bar{P}_{xDMF:yTHF}$ with respect to $A_{w}$ for a particular experimental humidity condition. Here E0 (1:3, wt) is at the boundary condition, as the fibre surface was with slight wrinkles; E1 (2:2, wt) is at the water-interaction condition, as the surface pores could be seen.

2) For THF, G1 is at the no-water-interaction condition, as the fibre surface was smooth; G0 is at the water-interaction condition, as the surface pores could be seen.

So in total,$0.02371\leq f\leq0.03557$, and$f=0.027$ is set in this experimental case.

**Figure 4a**

Relative humidity was maintained at e fixed range$RH=38\%\pm2\%$; the experiments were conducted within a fixed temperature range (25±2)°C (298.15K), thus the in-air water vapour temperature was supposed as 25°C (298.15 K).

For the in-air water vapour pressure, based on the Eq. 2

$$A_{w} (RH, 25^{\circ}C)\sim1.4\times RH\times\left( 298.15-273 \right)\times\left( 373-298.15 \right)$$

For DMF, the Antoine parameters are *A*=7.24128, *B*=1597.92, *C*=213.457

For THF, the Antoine parameters are *A*=7.10537, *B*=1256.68, *C*=232.621

For chloroform, the Antoine parameters are *A*=7.11148, *B*=1232.79, *C*=230.213

For acetone, the Antoine parameters are *A*=7.31414, *B*=1315.67, *C*=240.479

For ethyl acetate, the Antoine parameters are *A*=7.25963, *B*=1338.46, *C*= 228.608

Antoine Equation: $\log_{10}(P)=A-\frac{B}{C+T}$

Here *P* is the vapour pressure (unit in *mmHg*), *T* is the temperature (unit in °C); 1 *mmHg* =133.32237 Pa.

For DCM/DMF (3:1, v:v) mixtures, Raoult’s Law for ideal mixture of liquids was applied, taking co-solvent vapour pressure to be $\bar{P}_{xDCM:yDMF}=f(xP_{DCM}+yP_{DMF})$ , where *x:y* is the DCM to DMF molar ratio, we can evaluate $\bar{P}_{xDCM:yDMF}$ with respect to $A_{w}$ for a particular experimental humidity condition.

J0-J5 are all at the water-interaction condition, although J5 is at the boundary condition. So we have:

So in total,$f\leq0.01742$, and$f=0.017$ is set in this experimental case.

**Figure 4b**

Here the experiments were also conducted at room temperature, as stated in the article, so we choose 25°C as the experiment settings.

For the in-air water vapour pressure, based on the Eq. 2

$$A_{w} (RH, 25^{\circ}C)\sim1.4\times RH\times\left( 298.15-273 \right)\times\left( 373-298.15 \right)$$

For DMF, the Antoine parameters are *A*=7.24128, *B*=1597.92, *C*=213.457

Antoine Equation: $\log_{10}(P)=A-\frac{B}{C+T}$

Here *P* is the vapour pressure (unit in *mmHg*), *T* is the temperature (unit in °C); 1 *mmHg* =133.32237 Pa.

For DCM/DMF (v:v) mixtures, Raoult’s Law for ideal mixture of liquids was applied, taking co-solvent vapour pressure to be $\bar{P}_{xDCM:yDMF}=f(xP_{DCM}+yP_{DMF})$ , where *x:y* is the DCM to DMF molar ratio, we can evaluate $\bar{P}_{xDCM:yDMF}$ with respect to $A_{w}$ for a particular experimental humidity condition.

Here K0 is at the no-water-interaction condition, as the fibre surface was smooth; K2 is at the water-interaction condition, as the surface wrinkles could be seen, and K1 is at the boundary condition.

So in total,$0.0114\leq f\leq0.0275$, and$f=0.02$ is set in this experimental case.

**Figure 4c1**

Relative humidity was changing at different levels 30%-90%; the experiments were also conducted at different temperature levels 20-40°C.

For the in-air water vapour pressure, based on the Eq. 2

$$A_{w} (RH, 24^{\circ}C)\sim1.4\times RH\times\left( 297.15-273 \right)\times\left( 373-297.15 \right)$$

For choloroform, the Antoine parameters are *A*=7.11148, *B*=1232.79, *C*=230.213

Antoine Equation: $\log_{10}(P)=A-\frac{B}{C+T}$

Here *P* is the vapour pressure (unit in *mmHg*), *T* is the temperature (unit in °C); 1 *mmHg* =133.32237 Pa.

Here E4 is at the no-water-interaction condition, as the fibre surface was smooth; E5 is at the water-interaction condition, as the surface wrinkles could be seen.

So in total,$0.0498\leq f\leq0.0569$, and$f=0.05$ is set in this experimental case.

**Figure 4c2**

Relative humidity was changing at different levels 30%-90%; the experiments were also conducted at a fixed temperature 20°C.

For the in-air water vapour pressure, based on the Eq. 2

$$A_{w} (20^{\circ}C)\sim1.4\times RH\times\left( 293.15-273 \right)\times\left( 373-293.15 \right)$$

For chloroform, the Antoine parameters are *A*=7.11148, *B*=1232.79, *C*=230.213;

For THF, the Antoine parameters are *A*=7.10537, *B*=1256.68, *C*=232.621

Antoine Equation: $\log_{10}(P)=A-\frac{B}{C+T}$

Here *P* is the vapour pressure (unit in *mmHg*), *T* is the temperature (unit in °C); 1 *mmHg* =133.32237 Pa.

For chloroform/THF (wt/wt) mixtures, Raoult’s Law for ideal mixture of liquids was applied, taking co-solvent vapour pressure to be $\bar{P}_{xChloroform:yTHF}=f(xP_{chloroform}+yP_{THF})$ , where *x:y* is the chloroform to THF molar ratio, we can evaluate $\bar{P}_{xChloroform:yTHF}$ with respect to $A_{w}$ for a particular experimental humidity condition.

Here E4 is at the no-water-interaction condition, as the fibre surface was smooth; E5 is at the water-interaction condition, as the surface wrinkles could be seen.

So in total,$0.0356\leq f\leq0.0949$, and$f=0.05$ is set in this experimental case.
